# The Andersen Model of Total Patient Delay: a systematic review of its application in cancer diagnosis

**DOI:** 10.1258/jhsrp.2011.010113

**Published:** 2012-04

**Authors:** Fiona Walter, Andrew Webster, Suzanne Scott, Jon Emery

**Affiliations:** 1 General Practice and Primary Care Research Unit, Department of Public Health and Primary Care, University of Cambridge, Cambridge, UK; 2 General Practice, School of Primary, Aboriginal and Rural Health Care, University of Western Australia, Perth, Australia; 3 Department of Oral Health Services Research and Dental Public Health, King's College London, London, UK

## Abstract

**Objective:**

Patient pathways to presentation to health care professionals and initial management in primary care are key determinants of outcomes in cancer. Reducing diagnostic delays may result in improved prognosis and increase the proportion of early stage cancers identified. Investigating diagnostic delay could be facilitated by use of a robust theoretical framework. We systematically reviewed the literature reporting the application of Andersen's Model of Total Patient Delay (delay stages: appraisal, illness, behavioural, scheduling, treatment) in studies which assess cancer diagnosis.

**Methods:**

We searched four electronic databases and conducted a narrative synthesis. Inclusion criteria were studies which: reported primary research, focused on cancer diagnosis and explicitly applied one or more stages of the Andersen Model in the collection or analysis of data.

**Results:**

The vast majority of studies of diagnostic delay in cancer have not applied a theoretical model to inform data collection or reporting. Ten papers (reporting eight studies) met our inclusion criteria: three studied several cancers. The studies were heterogeneous in their methods and quality. The review confirmed that there are clearly identifiable stages between the recognition of a symptom, first presentation to a health care professional, subsequent diagnosis and initiation of treatment. There was strong evidence to support the existence and importance of appraisal and treatment delay as defined in the Andersen Model, although treatment delay requires expansion. There was some evidence to support scheduling delay which may be contributed to by both patient and the health service. Illness delay was often difficult to distinguish from appraisal delay. It was less clear whether behavioural delay exists as a separate significant stage.

**Conclusions:**

Greater consistency is required in the conduct and reporting of studies of diagnostic delay in cancer. We propose refinements to the Andersen Model which could be used to increase its validity and improve the consistency of reporting in future studies.

## Introduction

The UK has poorer cancer survival rates compared with other European countries with similar health care systems and expenditure.^1^ There is evidence that patient pathways to presentation, and initial management in the primary care setting, are key determinants of cancer patient outcomes.^2^ In the UK, patient delays in consulting health care professionals (HCPs) for symptoms and primary care delays in diagnosis contribute to a greater proportion of the time from the onset of symptoms to a definitive diagnosis than delays in referral or delays in starting cancer treatment.^3^ Reducing diagnostic delays may increase the proportion of early stage cancers identified, improve prognosis and reduce psychological distress.^4^ Current UK government policy (National Awareness and Early Diagnosis Initiative) supports raising public awareness of cancer symptoms, encouraging people to seek help earlier for these symptoms, and increasing the evidence base around diagnostic delay with the aim of improving clinical outcomes.

Most cancers are symptomatic (e.g. prostate 80%; breast and colorectal 75%),^5^ and most patients present these symptoms to their HCPs. However, cancer symptoms are the same as the symptoms of many other, more common, non-malignant conditions, and the vast majority of the time these symptoms do not herald serious diseases. To inform approaches to reduce diagnostic delay it is important to understand patient pathways to cancer diagnosis, including the timing and reasons behind the decision to seek help. A wide range of factors that directly or indirectly influence the time taken to seek help have been identified including: patient factors (e.g. age, gender), provider or system factors (e.g. access, patient-doctor communication), psychological factors (e.g. anxiety, fear), social factors (e.g. competing priorities as worker/carer) and behavioural factors (e.g. self-medication, watchful waiting).^6^

While there is a substantial literature on delays in cancer diagnosis, it suffers from a lack of consensus not only on the definitions and terms used, but also time intervals measured along the diagnostic pathway. This may be because most research into symptom appraisal and patient pathways has lacked a theoretical framework; these provide a systematic approach to improve understanding by building on existing knowledge and allowing hypothesis generation. Applying a theoretical framework to study delay in cancer diagnosis could underpin the development of future interventions to reduce patient time to presentation, diagnosis and cancer treatment.^7^

**Figure 1:**
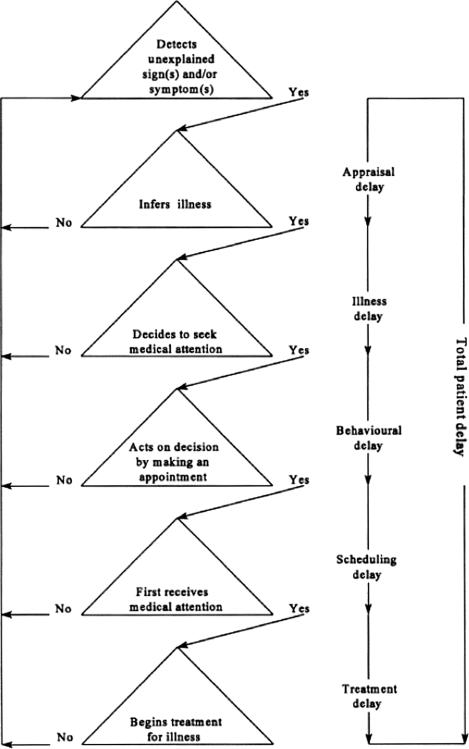
The General Model of Total Patient Delay as proposed by Andersen *et al.* (1995). Reproduced with permission from the British Journal of Social Psychology © The British Psychological Society^9^

Although few frameworks are used in cancer diagnostics research, the most widely cited theoretical model was first proposed by Safer *et al.*^8^ and subsequently developed by Andersen *et al*.^9^ Safer *et al.* proposed a three-stage model to account for the total time from first noticing a symptom to seeking treatment. ‘Appraisal delay’ described the time a person takes to evaluate a symptom as a sign of illness, ‘illness delay’ the time the person takes from the first sign of illness until deciding to seek professional medical care, and ‘utilization delay’ the time from the decision to seek care until the person consults a HCP. Andersen *et al.* presented a General Model of Total Patient Delay (‘the Andersen Model’) which could be applied to a variety of disorders. They conceptualized delay intervals occurring between phases of decision-making (the components of delay), and extended the model by replacing Safer *et al.*'s ‘utilization delay’ with ‘behavioural delay’ to describe the time between a person deciding an illness requires medical care and deciding to act on this decision; ‘scheduling delay’ the time between deciding to act on the decision to seek help and actually attending an appointment; and ‘treatment delay’ the time between the first appointment with a HCP and the onset of treatment (see Figure 1).

It is important to recognize subtle differences between Safer's original model and the Andersen Model: while Safer *et al.* used ‘stage’ to describe the delay time, Andersen *et al.* used ‘stage’ to describe not only the delay time but also the components of delay or phases of decision-making. Throughout this paper we shall apply the following terms for the sake of consistency: (a) ‘stage’ to describe the delay time, and (b) ‘components of delay’ to describe the decisional and appraisal processes that mark the beginning and end points of stages. Since its publication, the Andersen Model has been used to investigate delay in the diagnosis of many conditions such as myocardial infarction^10^ as well as cancer. This systematic review had two aims: first, to examine the application of the Andersen Model in studies which assess cancer diagnosis, and second, to assess the utility of the Andersen Model in conceptualizing and measuring the stages leading to cancer diagnosis.

## Methods

We performed systematic searches of the literature, followed by critical appraisal of included studies and a narrative synthesis.

### Systematic search

#### Data sources and search strategy

Our guiding definition to inform the initial search strategy was ‘published papers (quantitative and qualitative) which applied the Andersen Model during the collection and/or analysis of data in studies assessing cancer diagnosis’. We used a scoping exercise involving seven relevant papers to refine our search terms, and then conducted a systematic search of four electronic databases: PubMed, Web of Science, PsychINFO and International Bibliography of the Social Sciences (IBSS). The databases were searched from 1979 to July 2009, with no language restrictions. We also examined the reference lists and citations of all potentially relevant papers, and wrote to all first authors of included papers asking them about further relevant publications.

#### Inclusion and exclusion criteria (see Figure 2)

Our searches, including additional articles identified via references and citations, identified 13,392 abstracts, all of which were screened by one reviewer (AW); a second reviewer screened abstracts where it was unclear if the paper met the inclusion criteria (FW). Full text papers potentially for inclusion (*n* = 463) were assessed by at least two reviewers. All reviewers extracted data from the included papers using a standard proforma. Papers were excluded because they did not explicitly apply one or more stages of the Andersen Model in the collection or analysis of data, they did not focus on cancer diagnosis, or they did not report primary research. Regular consensus meetings with the reviewers ensured agreement on data extraction and interpretation of the data.

**Figure 2:**
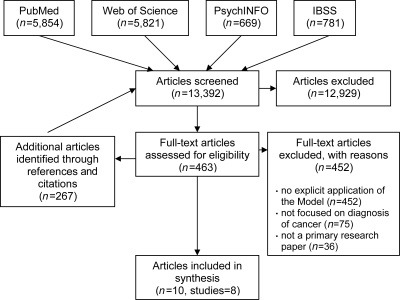
PRISMA Flow Diagram IBSS = International Bibliography of the Social Sciences

### Critical appraisal

Study quality was assessed at the same time as data extraction. The CASP (Critical Appraisal Skills Programme) checklists for qualitative and quantitative research were selected as they have been widely adopted: the checklists were slightly modified for our purposes. Mindful of controversies regarding critical appraisal,^11^ we used the CASP criteria as a guide to aspects of quality assessment, and adopted the approach of Dixon-Woods *et al.*^12^ where a paper is classified as a key paper, satisfactory, unsure, fatally flawed, or irrelevant. Each reviewer independently appraised each paper, and the overall rating was agreed by consensus.

### Narrative synthesis

Given the heterogeneity of studies, a narrative approach was deemed the most appropriate method as it uses text to summarize and explain the findings of the synthesis. This approach is increasingly used in policy research as a way of bringing together evidence from research conducted using a range of methods. As it has been criticized for a lack of transparency, we chose to increase the methodological rigour by following Rodgers *et al.*'s guidance on conducting narrative synthesis in systematic reviews:^13^ develop a theory, develop a preliminary synthesis, explore the relationships within and between studies, and assess the robustness of the synthesis product. The process of comparing and contrasting information across the included studies was facilitated by collating and tabulating the extracted data. These charts underpinned the discussion at consensus meetings. The main focus of the synthesis was a comparison of the included studies against the original conceptual framework provided by the Andersen Model. This comparison resulted in a mapping of ideas, key findings and methods against the original framework which highlighted commonalities and differences between and within the studies. Finally, we assessed the robustness of the synthesis by reviewing the primary data against the proposed revisions to the Model to ensure they remained consistent.

## Results

### Study and participant characteristics (see Table 1)

Ten papers were included; two studies were each reported in two separate papers.^14–17^ The studies were all published in English between 1995 and 2009 and were conducted in either western Europe or North America (Netherlands 3, UK 2, Spain 1, USA 1, Canada 1). Five studies used qualitative methods, two employed questionnaires (one self- and one researcher-administered), and one used a simulated patient approach. Three studies included more than one cancer site and the remainder studied a specific cancer site (breast, oral, larynx, rectum and ovary). No papers were excluded on the basis of methodological quality. Although all included papers were appraised as ‘key paper’ or ‘satisfactory’, the two ‘key paper’ studies^14,15,18^ demonstrated a more direct and complete application of the Andersen Model. Nonetheless, the CASP assessment highlighted widespread methodological flaws including: lack of specification of time intervals measured, lack of clarification of terms used or reporting of wording of questions used in interviews/questionnaires, and a reliance on retrospective accounts.

**Table 1:** Study and sample characteristics, methods, aims and appraisal of included studies

Study (***n* = 8)**	Country	Cancer site	Sample (***n*, gender, mean age/range)**	Method(s)	Aims	Appraisal*
de Nooijer *et al.* 2001 a & b	Netherlands	Breast, testicle, melanoma and colon	*n* = 23 57% Female 24–75 yrs	Retrospective interviews	To identify the stimulating and impeding factors influencing the transition to the next stage in the Andersen Model	KP
Brouha *et al.* 2005 a	Netherlands	Laryngeal	*n* = 117 22% Female ∼65 yrs**	Retrospective interviews	To determine the length of patient delay stages in patients with head and neck cancer and whether these delays were related to the stage of the disease at diagnosis	SAT
Brouha *et al.* 2005 b	Netherlands	Oral and pharyngeal	*n* = 189 36% Female 59 yrs	Retrospective interviews	As for Brouha 2005 a but patients with cancer at different sites	SAT
Dios *et al.* 2005	Spain	Oral	N/A ***	Descriptive study using simulated patients	To evaluate single delay phase – ‘*scheduling’ –* and to assess the influence of professional roles (receptionists) on this delay	SAT
Ristvedt and Trinkhaus 2005 and 2008	USA	Rectal	*n* = 69 40% Female 61 yrs	Retrospective questionnaire (self-completed)	To determine whether ‘trait anxiety’ is associated with the length of ‘*action appraisal*’ (a modification of Andersen's delay stages)	SAT
Bairati *et al.* 2006	Canada	Breast	120 Females 56 yrs	Administered questionnaire	To describe impeding and facilitating events in the process of the cancer care continuum in women with breast cancer	SAT
Evans *et al.* 2007	UK	Ovarian	43 Females 54 yrs	Retrospective interviews	To use accounts of provider delay from women with ovarian cancer to enhance the Andersen Model, and to suggest what GPs might do to minimize delays	SAT
Molassiotis *et al.* 2009	UK	Breast, brain, gastrointestinal, gynaecological, lung, head and neck	*n* = 74 49% Female 58 yrs	Retrospective interviews	To explore the pathway from initial persistent change in health to diagnosis of cancer in a sample of patients from seven cancer groups, and the factors mediating this process	KP

*KP = key paper; SAT = satisfactory

**Approximate- 59 participants were aged <65 yrs and 58 participants were aged >65 yrs

***Participant characteristics not reported as study used a simulated patient to call dental practices with two hypothetical complaints

### Interpretation and application of the Andersen Model (see Table 2, available online only at http://www.jhsrp.rsmjournals.com/cgi/content/full/jhsrp.2011.010113/DC1)

Table 2 shows that while some studies chose to focus their attention on a particular stage of the Andersen Model,^19,20^ others attempted to apply the Andersen Model more broadly,^14,15,18,21,22^ and one modified the definitions of the stages.^16,17^

Table 2 summarizes each study's interpretation of the stages and components of delay compared with the definitions given by Andersen *et al.*^9^ While some authors have interpreted the stages of delay exactly as defined,^21–23^ it is clear that many authors developed their own distinctive interpretations. For example, Molassiotis *et al.*^18^ defined ‘appraisal delay’ as ‘time from noticing change in health….’ compared with Andersen *et al.*'s definition of …‘time from when a person first detects an unexplained symptom…', a subtle but genuine change in meaning.

Table 2 also details the ways the studies applied and operationalized the Andersen Model. Five studies did not report the specific questions asked to identify the stages of delay,^18,20–23^ but most did give a general description of the question content. Three studies reported more specific details concerning the questions asked:^14,16,17,19^ for instance, de Nooijer *et al.* asked: ‘what symptoms did you detect? What was your explanation of the symptoms?’ compared with Ristvedt and Trinkaus's: ‘how long after your very first symptom did this occur?’ to assess appraisal delay.

### Key findings for specific components of delay (see Appendix 1, available online only at http://www.jhsrp.rsmjournals.com/cgi/content/full/jhsrp.2011.010113/DC1)

#### Appraisal delay

There was strong evidence confirming the existence and importance of appraisal delay in the patient pathway. The most important factor determining appraisal delay was the nature of the patient's symptoms. Brouha *et al.*^22^ reported that appraisal delay was longer among patients with pharyngeal cancer whose first symptoms were a sore throat and shorter in those with dysphagia or a neck mass. Among patients with oral cancer, appraisal delay was longer in those who attributed their symptoms to their prosthesis or dental problems and shorter in those with a painful lesion, irritation or dysphagia. De Nooijer *et al.*^14,15^ reported that patients with ‘red flag symptoms’ such as a breast lump, which were described as ‘known warning signs of cancer’, may not demonstrate a clear delay between detecting a symptom and inferring illness. They argued that in these cases appraisal delay did not appear to occur. In contrast, Ristvedt and Trinkhaus^16^ reported that some subjects sought medical attention for their symptoms while still believing that they were experiencing a benign condition. As the Andersen Model did not account for perceived seriousness, they developed their own concept of ‘symptom appraisal’, the time from symptom onset until recognition of seriousness or the visit to the doctor, whichever came first. Misattribution of symptoms, either to previously benign conditions (irritable bowel syndrome) or concurrent conditions (menopause, stress), or non-recognition of the seriousness of the symptoms, was found to make an important contribution to appraisal delay among women diagnosed with ovarian cancer.^22^ Other factors which influenced appraisal delay included the cancer site (significantly longer in those with glottic compared with non-glottic laryngeal tumours)^21^ and anxiety (males with low anxiety scores were more likely to experience increased appraisal time^17^). One study adapted the definitions of delay in order to include screen-detected patients with breast cancer.^23^

#### Illness delay

There was less evidence for the construct of illness delay as a separate entity to appraisal delay, although this may have been due to variations between studies in the interpretation and application of the Andersen Model. While illness delay did not appear to occur in a study of patients with laryngeal, oral or pharyngeal cancer, there were a significant number in each cancer site group who had inferred illness before deciding to seek help (27% laryngeal cancer,^21^ 35% pharyngeal cancer, 21% oral cancer).^22^ In contrast, a number of patients with symptoms of rectal cancer visited their doctor when still considering themselves to have a benign condition, and therefore illness delay did not occur (on the basis of their definition).^16,17^

#### Behavioural delay

There was limited evidence for the construct of behavioural delay; moreover, it appears that this stage may be minimal in length and therefore not a major component of delay. De Nooijer *et al.*^14,15^ reported that deciding to seek medical attention and acting on this decision cannot always be separated, so that in some instances patients did not experience behavioural delay. Where behavioural delay was identified, influences included competing events (e.g. holidays) and emotions (e.g. coming to terms with the meaning of symptoms). The two studies which only found behavioural delay in some cases may have been influenced by their interpretation and application of the Andersen Model: Brouha *et al.* asked patients why they had postponed medical consultation,^21,22^ and Bairati *et al.* did not report any specific questions to identify this stage of delay.^23^

#### Scheduling delay

There was conflicting evidence concerning the presence and importance of scheduling delay, with several studies suggesting that it is of short duration and limited importance. One study set out to specifically evaluate scheduling delay by using a simulated patient to telephone dental practices with a hypothetical complaint in order to determine the amount of time it would take for them to receive an appointment.^19^ They demonstrated its presence and, in contrast to the Andersen Model's assumption that scheduling delay is only caused by patients, showed the influence of other people and health care systems on scheduling delay for hypothetical oral cancer symptoms. Scheduling delays were found to be minimal in another study where only one woman cancelled her appointment for a medical consultation after receiving it, because she did not consider her symptoms to be serious enough.^18^ The extent and importance of scheduling delay may be under-represented as all the studies included in this review were conducted in affluent countries where patients have comparably good access to primary health care.

#### Treatment delay

There was strong evidence for the existence and importance of treatment delay. One study examined treatment delay among women with ovarian cancer, specifically from first presentation to diagnosis.^21^ Several factors were described as attributable, at least in part, to health care providers: non-investigation of symptoms (e.g. ignoring or dismissing menstruation-like pains in post-menopausal women), treatment for non-cancer causes (e.g. treating urinary incontinence with pelvic floor exercises), lack of follow-up to ensure resolution of symptoms, and referral delays (e.g. sending to incorrect medical speciality or non-urgently). The study found that delays attributed to patients are often compounded by doctors and the health system, though the authors noted that this may be a feature of ovarian cancer which is notoriously difficult to diagnose.

Several studies suggested expansions of treatment delay to include additional stages between ‘first receives medical attention’ and ‘begins treatment’. Bairati *et al.* investigated patients with breast cancer and identified a range of events which may occur after receiving medical attention: obtaining test results, obtaining a specialist appointment, having the medical consultation, obtaining the pathology report and onset of treatment. Again, health care providers and other organisations were shown to play an important role in facilitating or impeding treatment delay.^23^ Molassiotis *et al.* investigated patients with a range of cancers, and suggested two stages: from first consultation to diagnosis, and from diagnosis until treatment. The iterative nature of this process was also highlighted: one study reported that it may be possible for a return to earlier stages of delay via a (re)appraisal process following the initial medical consultation, in which a patient may again infer illness from their symptoms.^23^

## Discussion

This is the first systematic review of the application and utility of the Andersen Model in studying delays in cancer diagnosis and initiation of treatment. The UK's National Awareness and Early Diagnosis Initiative recognizes the importance of delays in presentation and diagnosis in determining cancer outcomes. At a time when international benchmarking studies are commencing to compare delay in cancer diagnosis, this review could inform the design and collection of data so that valid comparisons can be made across countries. Our searches identified a large number of studies examining diagnostic delay across different cancers, many of which were reviewed in full text as part of this systematic review. We only identified one other model from the literature which described three components of delay at patient, doctor and system levels.^24^ The vast majority of studies were atheoretical and applied a range of different definitions of delay and different methods to collect data. Consequently, there was no consistency of reporting delay across studies, making comparisons between cancer sites or health care systems extremely difficult.

We believe our intensive search strategies, and other standard approaches including contacting first authors and citation tracking, have identified all studies which have explicitly applied the Andersen Model to delayed diagnosis in cancer.^25,26^ We have applied explicit methods of data extraction and current best practice to narrative synthesis,^15^ including authors from different disciplinary perspectives. There were relatively few papers which met our inclusion criteria, and they were heterogeneous in their populations studied and overall methods. Nonetheless, comparing and contrasting their use of the Andersen Model provides sufficient evidence on which to make recommendations about its utility.

The key finding of our review is that there are clearly identifiable stages between the detection of a symptom, first presentation to a HCP, diagnosis and initiation of treatment. There is strong evidence to support the existence and importance of appraisal delay as defined in the Andersen Model. Illness delay is difficult to distinguish from appraisal delay: there are circumstances where patients may immediately interpret a symptom as being significant (e.g. breast lump) and therefore illness delay is imperceptible. Conversely, some people present symptoms to a HCP before recognizing they may represent illness. It is much less clear whether behavioural delay exists as a separate stage, contradicting the common assumption that the main reason for delayed help-seeking is denial. There is some evidence to support scheduling delay which may be contributed to by certain patient factors as well as their health care settings. Although there is evidence to support the existence and importance of treatment delay, it inadequately describes the steps between first presentation to a HCP leading to diagnosis and initiation of treatment. This process may involve several clinicians in the community and specialist setting, therefore incorporating delays in access to investigations as well as hospital care. While some studies have suggested that the Andersen Model could be simplified into fewer stages,^16,17^ others recommend an expansion of one or more stages of the Andersen Model.^20,23^

There was quite a large variation in the duration of total patient delay reported across studies. This may reflect methodological as well as tumour-specific differences, but for some patients and some symptoms ‘delay’ appears minimal. We therefore question the use of the term ‘delay’: it is not only value laden but also often inaccurate.^14^ It may be more appropriate to describe ***time intervals*** along the stages from symptom recognition to diagnosis and initiation of treatment. The review also revealed important differences in the way the Andersen Model was understood and applied leading to differences in definition, lack of specification of time intervals measured, and variation in wording used to ask patients about the different time intervals. There is a need for a model that can be consistently applied with clear definitions, not only of the time intervals, but also of the processes occurring during each stage. These stages need to have sufficient validity that they can be identified by patients, clinicians and researchers. This will allow the collection of comparable data between studies and across cancers.

**Figure 3:**
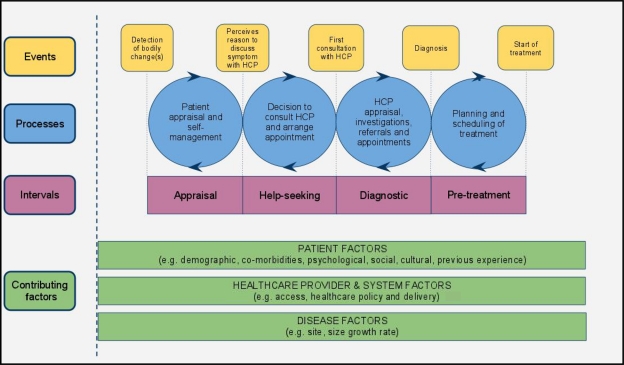
Model of pathways to treatment HCP = health care provider

Based on the findings of our review, we propose the refinements shown in Figure 3. First, we suggest that appraisal and illness delay are combined into the ‘appraisal interval’, with the start and end-points more clearly defined to describe the time interval from detection or awareness of a bodily change to perceiving a reason to discuss symptoms with a HCP; bodily changes will be appraised and responses other than seeking help (e.g. self-medication, self-monitoring) may be initiated. Second, we suggest combining behavioural delay with scheduling delay to become the ‘help-seeking interval’, describing the time interval from perceiving a reason to discuss symptoms with a HCP to the first consultation with a HCP about these symptoms. Third, the ‘diagnostic interval’ describes the time between first appointment with a HCP and the formal cancer diagnosis being made (acknowledging that, although in some cancer types the definitive diagnosis is only made after treatment, in the majority of cancers this event should be diagnosis at the multidisciplinary team meeting). This may involve referrals, several appointments and investigations, and, for some cancers, may involve a complex process. We have chosen not to break this interval down further into additional events within the pathway, but instead have incorporated these variations into the processes that occur within intervals. Fourth, the ‘pre-treatment interval’ describes the time between formal cancer diagnosis and initiation of treatment. We acknowledge that intervals between initiation of treatment and completion of treatment, including adjuvant modalities, can be an additional important contributor to outcome.^27^ A subsequent ‘treatment interval’ from the start of treatment to completion of treatment with curative intent could therefore be considered, but we believe this is beyond the scope of our proposed revisions given the original intent of the Andersen Model.

The revised model is generalizable across symptoms and across cancer sites; it is valid for symptoms which usually have a short appraisal interval (e.g. breast lump), and symptoms which often have very long appraisal intervals (e.g. prostatic symptoms, changing naevus). It is also generalizable across health care systems; those with well-developed primary care systems may have different processes during the diagnostic interval compared with systems with direct access to secondary care yet are likely to have similar events. The revised model encompasses the components of each interval by specifying the processes (within the blue circles), and their contributing factors (patient, health care provider and disease factors). The influence of these contributing factors also precedes the detection of bodily changes and extends beyond the start of treatment. Moreover, patients may not experience a linear passage through these intervals; instead, they may have periods of re-appraisal and re-scheduling following initial assessment by the HCP. We acknowledge that each of these time intervals requires more detailed description by symptom and disease group including the factors which shorten or prolong them: future unpacking of the processes will allow for deeper understanding which is likely to be cancer-site and population-specific. We also acknowledge that there is no particular start point, for instance, screen detected tumours may enter the pathway during the diagnostic interval. A major strength of this revised model is the identification of clear events that mark the beginning and end of each interval which can be identified by patients, clinicians and researchers. These revisions also address many of the conceptual issues that were raised in a recent discussion of the challenges of studying help-seeking behaviour.^28^

In conclusion, we believe future studies should explicitly apply a theoretical model to inform the measurement and description of time to cancer diagnosis and treatment initiation. This will result in greater consistency of reporting studies of diagnostic delay, allow better comparison of data across studies, build on existing knowledge, and in turn lead to more effective interventions. Our proposed model, which builds on the findings from this systematic review, could provide a useful theoretical approach for future studies of delays in diagnosis.
